# Correlation between upper gastrointestinal endoscopic findings and *Helicobacter pylori* detection in gastric biopsy specimens: a retrospective study

**DOI:** 10.11604/pamj.2026.53.12.49759

**Published:** 2026-01-12

**Authors:** Morenike Adedoyin Osundina, Hakeem Abiodun Alimi, Tajudeen Adeolu Badmos, Modupe Kehinde Ijikoyejo, Ifeanyichukwu Dupe Nwanji, Jesse Abiodun Otegbayo

**Affiliations:** 1 Gastroenterology Unit, Department of Medicine, University College Hospital, Ibadan, Nigeria,; 2 Pathology Department, University College Hospital, Ibadan, Nigeria

**Keywords:** *Helicobacter pylori*, gastric histology, upper gastrointestinal endoscopy, gastric erosions, peptic ulcer disease, gastric mucosa changes, gastric cancer

## Abstract

**Introduction:**

Helicobacter pylori (Hp) infection of the gastric mucosa has become a problem of public importance. HP inhabits the gastrointestinal tracts of greater than half of inhabitants of Africa, which is the highest recorded for any region of the world. It is a risk factor for gastric cancer. Hence, diagnosis and prompt treatment are imperative in forestalling the ill-effects. Prompt diagnosis and treatment are essential. Upper gastrointestinal endoscopy allows direct visualization of Hp-related mucosal changes. This study explores the correlation between endoscopic findings and histological confirmation of Hp infection.

**Methods:**

this retrospective, single-center descriptive study included all patients who underwent upper gastrointestinal endoscopy at the University College Hospital, Ibadan, Nigeria, from January 1, 2021, to December 31, 2023. Data collected included demographics, indications for gastroscopy, endoscopic findings, and gastric mucosal histopathology. Analysis was performed using SPSS version 20.

**Results:**

a total of 1,147 upper gastrointestinal endoscopies were performed during the study period, with 823 (71.7%) patients undergoing gastric biopsy. Of these, 377 (45.8%) were H. pylori-positive (Hp+), while 446 (54.2%) were negative (Hp-). Abnormal gastric and duodenal findings were significantly associated with Hp+ status, whereas normal mucosa correlated with Hp-status. Gastric erosions were more common in Hp+ patients (75.1% vs. 56.1%; χ^2^= 33.8, p < 0.001), indicating a potential link. The sensitivity of gastric erosions for detecting Hp infection was 75%, though specificity was below 50%.

**Conclusion:**

gastric erosions were more prevalent at gastroduodenoscopy and correlated well with the presence of H. pylori at histology.

## Introduction

*Helicobacter pylori* (*Hp*) is a non-sporulating, gram-negative bacterium. Morphologically, it can vary in shape from spiral, rod-shaped, curved-rod, coccoid, gull-winged, or U-shaped. It is microaerophilic and motile, the latter being made possible due to its embellishment with five to six sheathed lophotrichous flagella [1]. It was initially thought that the acidic milieu of the stomach made it a sterile organ. However, in the 1980s, Marshall and Warren demonstrated that *Hp* not only colonizes but also induces gastric inflammation [2]. *H. pylori* is considered a neutralophile, that is, capable of surviving between pH levels of 4 and 8, while growth is possible between pH 6 and 8 [3]. It is with these and other virulence factors that allow this organism to earn the status of a true resident of the gastric mucosa [4].

The global prevalence of *Hp* infection amongst both children and adults has decreased from 52.6% before 1990 to 43.9% in the years between 2015 and 2022. *Helicobacter pylori* inhabits the gastrointestinal tracts of more than half of the inhabitants of Africa, which is the highest recorded for any region of the world [5]. The infection is believed to be acquired during childhood and remains asymptomatic in many individuals, with only about 20 -30% becoming symptomatic. The transmission remains unclear, but oral-oral, faeco-oral, and gastric-oral modes have been identified as routes of infection, particularly in low socioeconomic households where poor sanitation and overcrowding drive the spread [6]. The deleterious effects of this organism on the gastroduodenal mucosa are well established, ranging from chronic gastritis, duodenitis, gastroduodenal ulcers, mucosa-associated lymphoid tissue lymphoma, and gastric cancer [7-9]. Additionally, it has been linked to a cascade of events that starts off as seemingly benign mucosal lesions culminating in a catastrophic malignant condition, in what is currently referred to in the literature as the Correa Cascade. While its effects have been well characterized in the stomach, research work has also been extended to evaluate potential links between *H. pylori* and other diseases in other organ systems, such as cardiometabolic, autoimmune diseases and even nutrient deficiencies, particularly vitamin B12 and iron [10,11]. It therefore implies that elimination of the organism from the gastrointestinal tract is imperative to forestall the dysfunctional epithelial barrier it incites on its way to inducing carcinogenesis.

Diagnosis of *Hp* could be via non-invasive or invasive techniques. Non-invasive methods include the urea breath test (UBT), serology, and stool antigen test. The invasive methods include the use of an endoscopic biopsy for rapid urease test (RUT), histology, culture, or polymerase chain reaction (PCR) [9,12-15]. Histology remains the gold standard for diagnosing *H. pylori*, providing direct insights into the presence and severity of the infection, as well as associated conditions like intestinal metaplasia, glandular atrophy, dysplasia, and neoplasia. Commonly used stains for detecting *H. pylori* include hematoxylin and eosin (HE), Giemsa, and Romanowsky. However, histology has its limitations, such as inter-observer variability in tissue assessment and the necessity of an endoscopy to obtain samples. Given the patchy distribution of *H. pylori* in the stomach, biopsies must be taken from multiple sites. The sensitivity and specificity of histology range from 53% to 90%, but these can be enhanced by increasing the number of biopsies and using specific stains [9]. Upper gastrointestinal endoscopy is a veritable tool used in obtaining gastric biopsies for the detection of *Hp*, either by culture, rapid urease test, or histological demonstration of the organism. Endoscopic features of *Hp* status have been documented in some studies, particularly in Caucasians and Asians. It is generally believed that regular arrangement of collecting venules (RAC) along the lesser curvature of the stomach is a marker of *Hp*-negative status, whereas findings such as diffuse redness, patchy/spotty redness, sticky mucus, antral nodularity, enlarged gastric folds, xanthoma, hyperplastic polyps, and gastric mucosa edema predict the presence of *Hp* infection [16].

Recent trends emphasize the direct diagnosis of *Helicobacter pylori* during endoscopy. Research has been conducted to ascertain whether mucosal changes can predict the presence of *H. pylori* infection or at least determine its absence, thereby guiding the decision to obtain biopsies for histological confirmation and avoiding delays in treatment. It has been demonstrated that enlarged gastric folds and antral nodularity predict *H. pylori* infection with a positive predictive value (PPV) of 100%, while fundic gland polyps and red streaks provide a 100% PPV for the exclusion of *H. pylori* infection using white light imaging during endoscopy. Furthermore, image-enhanced endoscopic techniques have shown superior sensitivity and specificity compared to white light imaging [16].

In the Kyoto classification, 5 endoscopic parameters (atrophy, enlarged folds, nodularity, diffused redness, and intestinal metaplasia) are scored with a minimum score of 0 and a maximum of 8. A score of 2 or more is associated with *Hp*-positive status, while scores ≥ 4 are associated with higher gastric cancer risk [17]. Whether these findings are enough to immediately initiate treatment is debatable. In resource-poor settings like ours, where histopathological turnaround time is longer, we seek to understand endoscopic features of *Hp* status in a simplified manner and whether the endoscopic appearance is enough to commence *Hp* eradication therapy. Unfortunately, information on endoscopic predictors of *Hp* status is sparse in Africa, with the most recent work done in Tanzania, where the presence of duodenal and gastric ulcers was the only statistically significant predictor of *Hp* presence [18]. This study aimed to correlate gastroduodenal endoscopic findings with the detection of *H. pylori* in histological examinations

## Methods

**Study design and setting:** this was a retrospective, descriptive cross-sectional study conducted at the University College Hospital (UCH), Ibadan, Oyo State, Nigeria. The study covered the period from 1 January 2021 to 31 December 2023. University College Hospital (UCH) is a tertiary referral center where upper gastrointestinal endoscopy is routinely performed by consultant gastroenterologists and gastroenterology trainees under the supervision of the consultants.

**Participants:** all patients who underwent upper gastrointestinal endoscopy within the study period were included.

**Variables:** the primary outcome was *Helicobacter pylori* infection status, determined by histological examination of gastric biopsy specimens. Exposure variables included endoscopic findings in the stomach and duodenum, recorded at the time of endoscopy (erosions, ulcers, polyps, masses, nodularity, haemorrhagic changes, and others). Additional variables extracted were demographic characteristics (age, sex) and indications for endoscopy (dyspepsia, upper gastrointestinal bleeding, dysphagia, recurrent vomiting, suspected malignancy, unexplained weight loss, and others).

**Data sources and measurement:** data were obtained from the endoscopy unit register and matched with pathology department records to retrieve histology results. Histology was regarded as the gold standard for *H. pylori* diagnosis. Endoscopic findings were documented prospectively at the time of the procedure using standard reporting formats, and the data were collected based on the findings at the time the procedures were done. Where multiple endoscopic abnormalities were present, each was recorded separately.

**Bias:** potential sources of bias included inter-observer variability in endoscopic reporting and missing histology results. Biopsies were routinely taken from the antrum even in patients with seemingly normal findings. However, some patients didn´t have biopsies taken. All procedures were performed by trained gastroenterologists or supervised trainees using a standardized reporting template.

**Study size:** no formal sample size calculation was performed. Instead, all eligible endoscopies performed during the study period were included, yielding 1,147 procedures, of which 823 (71.7%) had corresponding histology reports.

**Quantitative variables:** age was recorded as a continuous variable and expressed as mean ± standard deviation. For analysis, categorical variables such as sex, indications for endoscopy, and endoscopic findings were expressed as frequencies and percentages.

**Statistical methods:** data were analyzed using SPSS version 20 (IBM Corp., Armonk, NY, USA). Categorical variables were compared between *H. pylori*-positive and negative groups using the chi-square (χ^2^) test. Sensitivity and specificity of selected endoscopic findings (e.g., gastric erosion, any abnormal stomach or duodenum finding) for predicting *H. pylori* infection were calculated. A p-value <0.05 was considered statistically significant. Missing data were excluded from the analysis, particularly patients without a histological diagnosis.

**Ethical considerations:** ethical approval was obtained from the Joint University of Ibadan/University College Hospital Ethics Committee (UI/EC/24/0845). Patient confidentiality was maintained by anonymizing all data during extraction and analysis.

## Results

**Participants:** a total of 1,147 upper gastrointestinal endoscopic procedures were performed during the study period. Histological reports were available for 823 cases in which antral biopsies were obtained. The remaining procedures had no histological results of gastric antral biopsy, which is routinely performed to identify *Helicobacter pylori* infection even in endoscopically normal mucosa.

**Descriptive data:** out of the 1,147 individuals who had the procedure done, 609 (53.2%) were females, and 535 (46.8%) were males, with an average age of 51.4±16.7 years. The most prevalent indication for endoscopy was dyspepsia in 642 (56.1%), followed by upper gastrointestinal bleeding in 163(14.2%) and dysphagia in 65 (5.7%).

**Figure 1:**
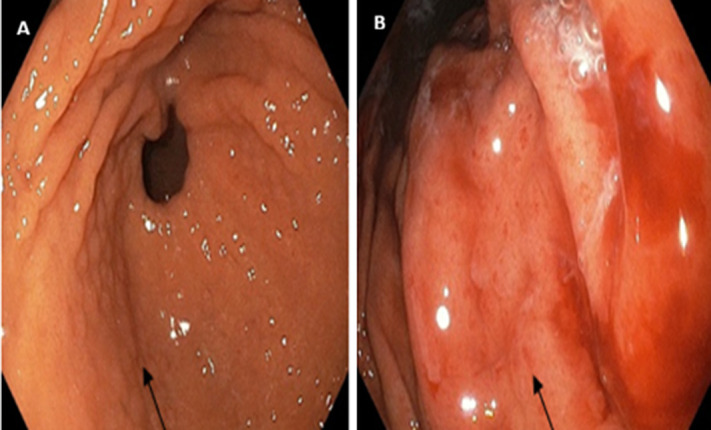
endoscopic images of some patients who had *Helicobacter pylori* confirmed by histology: A) revealed diffused nodularity; B) had features of haemorrhagic gastritis (black arrows in A and B, respectively)

**Figure 2:**
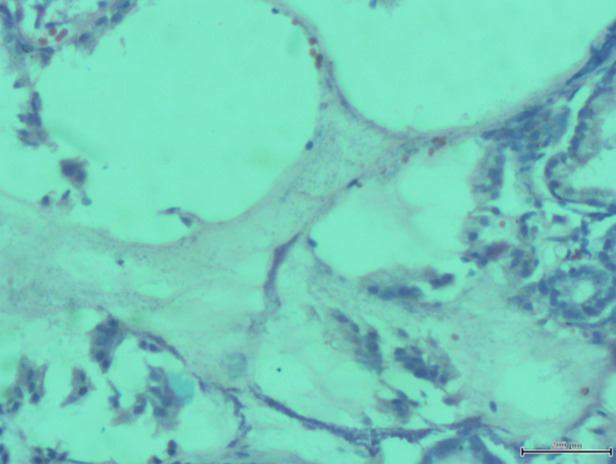
photomicrograph of the stomach antrum demonstrating florid colonization of the surface of the mucous layer by short rod-shaped bacilli (Giemsa x400)

**Outcome data:** the commonest endoscopic finding that correlates with the presence of *Hp* was gastric erosions. Other less common findings were antral nodularity and haemorrhagic gastritis, as shown in Figure 1, while Figure 2 is one of the antral biopsy histology showing *Hp*-like organisms.

**Main results:** histology was traceable in 823 (71.7%) procedures. 377 (45.8%) had *H. pylori* detected (*Hp*+) at histology, and 446 (54.2%) without (Hp-). The mean age for those with Hp+ status was 51.1 ± 16.0, while it was 52.5 ± 15.9 for *Hp*- status. The gender distribution as well as the indication for upper GI endoscopy were similar in both groups. Indications and endoscopic findings based on *Hp* status are shown in Table 1, Table 2, Table 3.

**Table 1:** gender distribution and clinical indications by *H. pylori* status

Variable	*HP*-negative (N=446)	*HP*-positive (N=377)	χ^2^ (p-value)
**Gender**			
Female	249 (55.8%)	211 (56.0%)	
Male	197 (44.2%)	166 (44.0%)	0.0 (0.968)
**Indications**			
Dyspepsia	263 (59.0%)	247 (65.5%)	3.7 (0.056)
Upper GI bleeding	59 (13.2%)	40 (10.6%)	1.3 (0.253)
Dysphagia	21 (4.7%)	17 (4.5%)	0.0 (0.892)
Recurrent vomiting	16 (3.6%)	7 (1.9%)	2.1 (0.142)
Suspected malignancy	15 (3.4%)	11 (2.9%)	0.2 (0.684)
Unexplained weight loss	9 (2.0%)	6 (1.6%)	0.2 (0.669)
Others	63 (14.1%)	49 (13.0%)	0.2 (0.647)

**Table 2:** gastric endoscopic findings by *H. pylori* status

Stomach findings	*HP*-negative (N=446)	*HP*-positive (N=377)	χ^2^ (p-value)
Erosions	248 (55.6%)	283 (75.1%)	33.9 (0.000)*
Ulcer	43 (9.6%)	28 (7.4%)	1.3 (0.262)
Mass	32 (7.2%)	13 (3.4%)	5.5 (0.017)*
Polyps	24 (5.4%)	13 (3.4%)	1.9 (0.167)
Others	1 (0.2%)	17 (4.5%)	17.8 (0.000)*
Normal	94 (21.1%)	23 (6.1%)	37.6 (0.001)*

* Statistically significant

**Table 3:** duodenal findings and other endoscopic features by H. pylori status

Duodenal findings	*HP*-negative (N=446)	*HP*-positive (N=377)	χ^2^ (p-value)
Erosions	48 (10.8%)	47 (12.5%)	0.6 (0.448)
Ulcer	15 (3.4%)	13 (3.4%)	0.0 (1.000)
Mass	9 (2.0%)	1 (0.3%)	4.9 (0.027)*
Polyps	6 (1.3%)	1 (0.3%)	2.4 (0.118)
Lymphangiectasia	10 (2.2%)	7 (1.9%)	0.1 (0.763)
Others	6 (1.3%)	10 (2.7%)	2.1 (0.147)
Normal	348 (78.0%)	298 (79.0%)	0.1 (0.728)
Duodenogastric reflux	18 (4.0%)	14 (3.7%)	0.0 (0.824)
Normal endoscopy	24 (5.4%)	6 (1.6%)	8.4 (0.003)*

* Statistically significant

**Other analysis:** the sensitivity of gastric erosion was 75.1%, while erosions in the duodenum had a sensitivity of 89.2%. Diagnostic performance of any abnormality in the stomach and duodenum, as shown in Table 4, while Table 5 shows the summary of histological reports of patients with *Hp-negative* status.

**Table 4:** diagnostic performance of endoscopic findings in predicting *H. pylori*-positive status

Parameter	Sensitivity (%)	Specificity (%)
Gastric erosion	75.1	44.4
Any gastric abnormality	93.9	22.0
Duodenal erosion	12.5	89.2
Any duodenal abnormality	21.0	66.8
Abnormal endoscopy	98.4	5.4

**Table 5:** histological findings in gastric biopsies of H. pylori-negative patients

Histological finding	Frequency (%)
Chronic active gastritis	154 (34.5%)
Chronic inactive gastritis	86 (19.2%)
Chronic non-specific gastritis	31 (7.0%)
Intestinal metaplasia	14 (3.1%)
Chronic atrophic gastritis	13 (2.9%)
Mass lesions - adenocarcinoma	23 (5.1%)
Mass lesions - squamous carcinoma	1 (0.2%)
Mass lesions - non-malignant	10 (2.2%)
Inflammatory polyps	9 (2.0%)
Peutz-Jeghers polyp	1 (0.2%)
Hyperplastic polyps	6 (1.3%)
Polyps reported as gastritis	7 (1.6%)

## Discussion

In this study, abnormal findings in the stomach and duodenum were strongly associated with *Hp*-positive status, while normal findings either in the gastric or duodenal mucosa were associated with *Hp*-negative status. The prevalence of *H. pylori* diagnosed histologically was 46%, which was higher than the 44.1% reported by Mao *et al*. [19], 26% reported in Japan [20], and 14.16% in Lebanon [21]. Only 6% of patients with *Hp* had normal gastric epithelium on white light endoscopy, which was considerably lower than the 20 percent reported in a different study [16]. This difference may be because the latter study reported a lower prevalence, despite using advanced image-enhanced endoscopy techniques. The sensitivity of gastric erosion observed in our study was 75%, whereas the specificity was significantly lower than 50%. This finding is in contrast to previous research that demonstrated low sensitivity but high specificity of gastric erosion for *Hp* infection, although those studies questioned the clinical utility of gastric erosion as a predictor of *Hp* status [19,20,22]. This discrepancy may be attributed, in part, to variations in the reporting methods of pathological gastric findings. Additionally, our study revealed a notably high prevalence of gastric erosions, even among *Helicobacter pylori*-negative patients, at a rate of 55.6%.

There were significant differences in endoscopic findings between patients with *Hp*-positive (*Hp*+) and *Hp*-negative (*Hp*-) histology results. In the stomach, erosions were more frequently observed at endoscopy in patients with histologically confirmed *H. pylori* infection (75.1% vs 56.1%), suggesting that the presence of this abnormality may be associated with *H. pylori* infection (χ^2^= 33.8, p < 0.001). This is consistent with previous research and aligns with the well-documented role of *Hp* in gastric mucosal pathology [19]. This underscores the potential for endoscopy to reveal mucosal abnormalities linked to *Hp* infection, thereby providing valuable diagnostic information [20,23].

In contrast to the findings in the stomach, there appears to be a similar pattern for both groups in their endoscopic duodenal findings. This is a less common finding and warrants further investigation [24]. These results suggest that while *Hp* infection is a key factor in gastric pathology, its role in duodenal conditions may be less pronounced or influenced by other factors. This is particularly evident when the sensitivity of any abnormality in the gastric mucosa (93.9%) and duodenal mucosa (21.0%) is compared. This finding is similar to what was found among the Lebanese cohort, where less than a third had inflammatory changes in the duodenum, though this reached statistical significance, unlike in our study [21]. Unlike this study where there was an attempt to characterize duodenal mucosa changes, some similar studies did not document the endoscopic appearance of the duodenal mucosa as an indicator of *Hp* status. [19,20].

Furthermore, there were more histologically confirmed gastric cancer cases in the *Hp*-negative group. Sampling of mass lesions without adjacent areas may explain this finding. Another plausible explanation is bias in reporting; histopathological findings of a cancer may lead to under-reporting of *H. pylori*. In addition, the hit-and-run hypothesis of *Hp* carcinogenesis has been well described in the literature [25-27]. The theory proposes that long-standing infection-induced carcinogenesis, but continuous presence of the organism in the stomach is not required once gastric cancer is established. Nevertheless, 3.9% (45/1147) of all patients who had upper gastrointestinal endoscopy done had gastric masses, and about a third had *Hp*-associated gastritis. However, it has been shown that genomic techniques can increase the identification of *Hp* in gastric cancer tissue, with 89% reported for Latin America and 85% in Europe in the Legacy study, and we may benefit from this in our region [28]. Our findings highlight the importance of integrating endoscopic and histological evaluations for diagnosing *H. pylori*. Gastric erosions observed during endoscopy should raise suspicion of *Hp* infection, given their strong association with *Hp* infection in our study. Histological confirmation, however, remains essential due to the varied presentation of *H. pylori* infection.

**Limitation:** while the Kyoto classification was used in most studies assessing endoscopic predictors of *Hp* infection, this was not the case in this work. While our study provides significant insights, it is limited by the retrospective nature and the single-centre setting, which may affect the generalizability of the findings. Future research could make use of a multicentric approach and a prospective study design to validate our results.

## Conclusion

Gastric abnormalities were more prevalent at endoscopy, and gastric erosion was well correlated with the histological diagnosis of *H. pylori*. Therefore, empiric eradication therapy could be instituted in this category of patients, particularly in resource-poor settings like ours.

### What is known about this topic

- H. pylori infects more than half of Africa´s population, the highest regional prevalence worldwide;
- Endoscopic features (e.g., gastric erosions, nodularity, enlarged folds) have been linked to H. pylori infection, mainly in Asian and Western cohorts;
- African data on endoscopic predictors of H. pylori infection is sparse; the most recent work (Tanzania) found ulcers as the only predictor.

### What this study adds

- Gastric erosions were strongly associated with H. pylori infection;
- Abnormal gastric mucosa had very high sensitivity (93.9%) for H. pylori positivity, while normal findings were linked with negative status;
- These findings suggest that in resource-poor settings, patients with gastric erosions or abnormal mucosa on endoscopy may reasonably be started on empiric eradication therapy while awaiting histology.

## References

[1] Umar Z, Tang JW, Marshall BJ, Tay AC, Wang L (2025). Rapid diagnosis and precision treatment of *Helicobacter pylori* infection in clinical settings. Crit Rev Microbiol.

[2] Marshall B, Warren JR (1984). Unidentified curved bacilli in the stomach of patients with gastritis and peptic ulceration. The Lancet.

[3] Marcus EA, Scott DR (2024). Gastric colonization by H. *pylori* In Helicobacter pylori Singapore: Springer. Nature Singapore.

[4] Spagnuolo R, Scarlata GG, Paravati MR, Abenavoli L, Luzza F (2024). Change in diagnosis of Helicobacter pylori infection in the treatment-failure era. Antibiotics.

[5] Chen YC, Malfertheiner P, Yu HT, Kuo CL, Chang YY, Meng FT (2024). Global prevalence of Helicobacter pylori infection and incidence of gastric cancer between 1980 and 2022. Gastroenterology.

[6] Balyorugulu G (2024). Helicobacter Pylori in Children. African Journal of Gastroenterology and Hepatology.

[7] Reyes VE (2023). Helicobacter pylori and its role in gastric cancer. Microorganisms.

[8] Tohumcu E, Kaitsas F, Bricca L, Ruggeri A, Gasbarrini A, Cammarota G (2024). *Helicobacter pylori* and the human Gastrointestinal microbiota: A multifaceted relationship. Antibiotics.

[9] Garza-González E, Perez-Perez GI, Maldonado-Garza HJ, Bosques-Padilla FJ (2014). A review of *Helicobacter pylori* diagnosis, treatment, and methods to detect eradication. World Journal of Gastroenterology: World J Gastroenterol.

[10] Ali A, AlHussaini KI (2024). *Helicobacter pylori* a contemporary perspective on pathogenesis, diagnosis and treatment strategies. Microorganisms.

[11] Hashim ER, Abufaddan NH, Osman AM, Medhat MA (2024). *Helicobacter pylori* Infection in Children: An Uphill Climb. Afro-Egyptian Journal of Infectious and Endemic Diseases.

[12] Ferwana M, Abdulmajeed I, Alhajiahmed A, Madani W, Firwana B, Hasan R (2015). Accuracy of urea breath test in *Helicobacter pylori* infection: meta-analysis. World J Gastroenterol.

[13] Mohammadian T, Ganji L (2019). The diagnostic tests for detection of *Helicobacter pylori* infection. Monoclonal antibodies in immunodiagnosis and immunotherapy.

[14] Uotani T, Graham DY (2015). Diagnosis of *Helicobacter pylori* using the rapid urease test. Ann Transl Med.

[15] Sulo P, Šipková B (2021). DNA diagnostics for reliable and universal identification of *Helicobacter pylori*. World J Gastroenterol.

[16] Chatrangsun B, Aumpan N, Pornthisarn B, Chonprasertsuk S, Siramolpiwat S, Bhanthumkomol P (2024). Simultaneous detection of *Helicobacter pylori* infection comparing between white light and image-enhanced endoscopy. BMC gastroenterology.

[17] Toyoshima O, Nishizawa T (2022). Kyoto classification of gastritis: Advances and future perspectives in endoscopic diagnosis of gastritis. World J Gastroenterol.

[18] Muhina IAI, Sadiq AM, Said FH, Raza FM, Gharib SK, Muhali SS (2024). Feco-prevalence, endoscopic pattern and associated factors of *Helicobacter pylori* infection among symptomatic adult patients in Northern Tanzania. PLoS One.

[19] Mao T, Wang Y, Yin F, Zhao Q, Yang L, Ding X, Tian Z (2016). Association of endoscopic features of gastric mucosa with *Helicobacter pylori* infection in Chinese patients. Gastroenterol Res Pract.

[20] Study Group for Establishing Endoscopic Diagnosis of Chronic Gastritis (2013). Diagnosis of *Helicobacter pylori* infection in gastric mucosa by endoscopic features: A multicenter prospective study. Dig Endosc.

[21] Hind J, Bilal A, Rania I, Walid N, Sara M (2025). Assessment of *Helicobacter pylori* infection in Lebanon: endoscopic and histopathological findings. J Infect Public Health.

[22] Gonen C, Simsek I, Sarioglu S, Akpinar H (2009). Comparison of High Resolution Magnifying Endoscopy and Standard Videoendoscopy for the Diagnosis of *Helicobacter pylori* Gastritis in Routine Clinical Practice: A Prospective Study. Helicobacter.

[23] Cho JH, Chang YW, Jang JY, Shim JJ, Lee CK, Dong SH (2013). Close observation of gastric mucosal pattern by standard endoscopy can predict *Helicobacter pylori* infection status. J Gastroenterol Hepatol.

[24] Boixeda D, Gisbert JP, Martín de Argila C, Cantón R, al-Mustafa A (1995). H. pylori infection at the duodenal bulb in different endoscopic diagnoses. Rev Clin Esp.

[25] Hatakeyama M (2014). *Helicobacter pylori* CagA and gastric cancer: a paradigm for hit-and-run carcinogenesis. Cell Host Microbe.

[26] Lin AS, McClain MS, Beckett AC, Caston RR, Harvey ML, Dixon BREA (2020). Temporal Control of the *Helicobacter pylori* Cag Type IV Secretion System in a Mongolian Gerbil Model of Gastric Carcinogenesis. mBio.

[27] Takahashi-Kanemitsu A, Knight CT, Hatakeyama M (2020). Molecular anatomy and pathogenic actions of *Helicobacter pylori* CagA that underpin gastric carcinogenesis. Cell Mol Immunol.

[28] Martínez-Ciarpaglini C, Barros R, Caballero C, Boggino H, Alarcón-Molero L, Peleteiro B (2025). Comprehensive histopathological analysis of gastric cancer in European and Latin America populations reveals differences in PDL1, HER2, p53 and MUC6 expression. Gastric Cancer.

